# Managing uncertainty: a review of food system scenario analysis and modelling

**DOI:** 10.1098/rstb.2010.0141

**Published:** 2010-09-27

**Authors:** Michael Reilly, Dirk Willenbockel

**Affiliations:** 1 Foresight Research and Knowledge Management, Government Office for Science, 1 Victoria Street, London SW1H 0ET, UK; 2 Institute of Development Studies at the University of Sussex, Brighton, UK

**Keywords:** food system, scenarios, models, modelling, futures, integrated assessment

## Abstract

Complex socio-ecological systems like the food system are unpredictable, especially to long-term horizons such as 2050. In order to manage this uncertainty, scenario analysis has been used in conjunction with food system models to explore plausible future outcomes. Food system scenarios use a diversity of scenario types and modelling approaches determined by the purpose of the exercise and by technical, methodological and epistemological constraints. Our case studies do not suggest Malthusian futures for a projected global population of 9 billion in 2050; but international trade will be a crucial determinant of outcomes; and the concept of sustainability across the dimensions of the food system has been inadequately explored so far. The impact of scenario analysis at a global scale could be strengthened with participatory processes involving key actors at other geographical scales. Food system models are valuable in managing existing knowledge on system behaviour and ensuring the credibility of qualitative stories but they are limited by current datasets for global crop production and trade, land use and hydrology. Climate change is likely to challenge the adaptive capacity of agricultural production and there are important knowledge gaps for modelling research to address.

## 1. Introduction

The first documented attempt to use modelling to explore the uncertainty surrounding the world's ability to feed a growing population was possibly by Malthus (1798) in the first edition of *An Essay on the Principles of Population*. This essay famously put forth the hypothesis that exponential population growth and its associated demand for food would overwhelm linear growth of supply. Malthus's hypothesis has been subject to persistent challenge both empirically and theoretically (Boserup 1965); but with population growth projected by the UN to increase to 9.1 billion in 2050, concerns remain.

More recently, according to McCalla & Revoredo (2001), there have been at least 30 different major long-term model-based simulations of global food supply and demand undertaken over the second half of the twentieth century. In the past decade, a number of further studies concerned with the future of the global food system have been published. In order to manage the uncertainties inherent in this system, these studies have used scenario analysis as well as model simulations. This article reviews a selection of contemporary studies which use scenario analysis and modelling to explore the future of the global food system to 2050. The case studies under review are World Agriculture towards 2030/2050, the Comprehensive Assessment of Water Management in Agriculture (CAWMA), a study on the effects of climate change on global food production based on Intergovernmental Panel on Climate Change socio-economic scenarios, the Millennium Ecosystem Assessment (MA) scenarios and the Agrimonde 1 scenario. Case studies have been chosen to illustrate a scenario typology and to demonstrate a diversity of modelling approaches.

We begin with a short history of scenario analysis and then provide an explanation of why scenarios have been increasingly used to manage uncertainty in systems with socio-economic and biophysical dimensions. A typology is proposed to classify the scenarios used by the case studies. An outline of the quantitative modelling approaches of the case studies is followed by brief summaries of their scenarios and a discussion of results. The article concludes by considering some of the challenges for food system scenario analysis and modelling.

## 2. Scenario analysis

### (a) Origins of scenario analysis

The Oxford English Dictionary defines a scenario as ‘*a postulated sequence or development of events*’. Prominent proponents of scenario analysis view scenarios variously as ‘hypothetical sequences of events constructed for the purpose of focusing attention on causal processes and decision-points’ (Kahn & Wiener 1967, p. 6),*‘*focused descriptions of fundamentally different futures presented in a coherent script-like or narrative fashion*’* (Schoemaker 1993, p. 195), *‘*internally consistent and challenging narrative descriptions of possible futures*’* (van der Heijden 2005, p. 14), as ‘a tool for ordering one's perceptions about alternative future environments in which one's decisions might be played out’ (Schwartz 1991, p. 4) or ‘a description of potential future conditions, developed to inform decision-making under uncertainty’ (Parson *et al*. 2007, p. 1).

The emphasis on exploring multiple futures underlines that scenario analysis does not aim to predict the future. Scenario analysis copes with uncertainty by presenting a range of plausible futures, usually without assigning probabilities to the outcomes. In particular, for complex socio-ecological systems, scenarios can be used to explore uncertainties over long-term horizons that cannot be represented by probability distributions on known parameters (Swart *et al*. 2004; Parson 2008).

The origins of scenario analysis lead back to the Manhattan Project in 1942, where the limits of using probability in decision-making led to computer simulations of atomic explosions. The concept was further refined after World War II at the RAND Corporation, particularly by Hermann Kahn, and especially for the large-scale early warning system Air Defence System Missile Command. Kahn's book *On Thermonuclear War* used scenario analysis to explore the uncertainties surrounding nuclear war (Kahn 1960). In 1961, Kahn left RAND to set up the Hudson Institute, a think-tank with a broader remit for scenario analysis. A subsequent book *The Year 2000* written in 1967 graduated his methods beyond military planning; and it was also a signal of growing curiosity in the comparative advantage scenario analysis might offer to business.

Pierre Wack pioneered the use of scenario analysis at Shell based on possibilities presented by Kahn for corporate planning. In the late 1960s, Shell used a system of Unified Planning Machinery with a 6 year horizon to prepare its value chain for the future. It was posited however, on a single ‘business as usual’ scenario. Wack participated in an experiment to look ahead 15 years in an exercise called Horizon Year Planning. The striking findings of the study, which suggested that transformative change could be imminent in the oil market, provoked Shell in 1971 to migrate from predictive forecasting to a new method of scenario analysis (Wack 1985*a*).

The approach employed by Wack at Shell, and adapted from Kahn's early work, identifies predetermined elements in a system of interest in order that the outcomes of uncertainties, which are prioritized strategically, can be explored in multiple scenarios.

### (b) How scenario analysis can be used to manage uncertainty

The food system shares an important attribute with that of the energy system: crop-based technologies often have long lead times. Strategic planning is likely to become increasingly necessary if the world is to feed a projected 9 billion people healthily and sustainably in 2050.

The food system is multi-dimensional (Ericksen 2008) and includes social, economic, biophysical, political and institutional dimensions. Using a model as a proxy to this system raises ontological and epistemological issues (Rotmans & van Asselt 2001). Funtowicz & Ravetz (1990) suggest three types of uncertainties in integrated assessment:

— technical uncertainties;
— methodological uncertainties;
— epistemological uncertainties.

There are technical uncertainties concerning the quality of data available to calibrate the model and to determine input assumptions for drivers of change. There are methodological uncertainties because we may lack sufficient knowledge to create an adequate model form with suitable structure and functional forms of behavioural equations. Epistemological uncertainties refer to the completeness of the model: changes in human behaviour and values, randomness of nature, technological surprises and so-called high impact, high uncertainty ‘black swan’ events may all be unknowable (Taleb 2007). Furthermore, a complex system may be fundamentally indeterminate. An accumulation of these uncertainties in a model simulation makes assigning probabilities to outcomes challenging.

These challenges notwithstanding, scenario analysis offers an opportunity to manage technical uncertainty in the socio-economic dimensions of the food system differently from uncertainties in its biophysical dimension (Rotmans & van Asselt 2001; Döös 2002). Model simulations using scenarios of multiple input assumptions for socio-economic variables may mitigate technical uncertainties in the model. However, it will not be robust to manage uncertainty through multiple input assumptions for socio-economic variables if uncertainties in its biophysical dimensions are not respected. For example, current models used to simulate the effects of climate change on sea-level rise may not be adequate proxies to the system because of epistemological uncertainties surrounding the dynamics of melting ice sheets (Hansen 2007). In addition, it will not be accurate to quantify socio-economic drivers of change as discrete or exogenous if they are actually endogenous to the system or correlated with other drivers (Garb *et al*. 2008).

### (c) Typology of scenarios

If scenarios are to be used to manage the uncertainties that can accumulate in models, the type of scenario chosen will depend on the purpose of the exercise. A typology modified from Börjeson *et al*. (2005) is proposed to classify three different approaches to scenarios of the future:

— projections;
— exploratory scenarios;
— normative scenarios.

Baseline projections can be used to estimate the future state of a system subject to ‘business as usual’ assumptions with no major policy changes. Projections can also be used to pose the question of how a system reacts if a certain set of ‘what-if’ assumptions are made. Such scenarios, which quantify outcomes, are challenged by uncertainty in the long term, and may not explore adequately variations in socio-economic drivers, or transformation in the system (Alcamo 2001). On the other hand, the process to create projections is likely to be less time-consuming than for other scenario types; and they may have utility to food system actors.

The narratives of exploratory scenarios are predominantly qualitative but usually with a quantitative underpinning provided by model simulation outputs. They can either focus on drivers of change that are exogenous to the system and out-with the control of the actors for whom the scenarios are being developed, external scenarios, or they can include policy, in which case they are described as strategic. Exploratory scenarios are useful if the uncertainties in the system cannot be sufficiently managed using a model, or modelling framework alone. For example, a technological surprise like the ‘green revolution’ would have been very difficult to simulate using prior historical data but nonetheless had a profound impact on the food system and its outcomes (Evans 1998). Methodological and epistemological uncertainty may be explored using qualitative narratives.

Normative scenarios develop stories that meet specific outcomes or targets. Preserving scenarios seek out pathways for the system to reach an outcome without transformation. Alternatively, transforming scenarios assume that change in the system will be necessary to meet the normative target. Although normative scenarios meet a specific outcome or target, they are, paradoxically, the least predictive of scenario types. Indeed, such scenarios may be helpful in reducing dilemmas of legitimacy in futures analysis (Robinson 1992).

## 3. Food system modelling in scenario case studies

The scenario studies included in this review use models of the food system to simulate endogenous variables including food production and consumption. Table 1 provides a brief synopsis of the various models employed, distinguishing the key variables determined endogenously by each model from drivers of change that are exogenous to the model and based on external assumptions. The geographical and sectoral resolution of the models is also provided.

**Table 1.** Main simulation models used in the scenario studies.

model (affiliation)	type	scenario study	main endogenous variables	main exogenous drivers	spatial scale	sectoral scale	documentation
IMAGE 2.2 (RIVM)	integrated assessment	MA	energy use, land use, GHG emissions, climate	population, GDP	17 regions, biophysical: 0.5^o^*0.5^o^ grid	12 agric. commodities	IMAGE (2001)
AIM (NIES/Kyoto University)	integrated assessment	MA	land cover, emissions, water use (Asia Pacific)	population, productivity	15 regions, Water: 2.5^o^*2.5^o^ grid	15 production sectors (4 agric. sectors)	Kainuma *et al*. (2002)
IMPACT (IFPRI)	multi-market partial equilibrium	MA	agricultural production, demand, prices and trade, child malnutrition	population, GDP, agric. productivity	43 regions	32 agric. commodities	www.ifpri.org
WaterGAP (University of Kassel)	hydrology	MA	water use, water stress	population, GDP, climate, land cover	150 regions water: 0.5^o^*0.5^o^ grid	n.a.	Alcamo *et al*. (2003)
ECOPATH/ECOSIM (University of British Columbia)	biophysical	MA	marine ecosystem, biomass	marine species mortality, fishery catch	flexible	n.a.	Christensen & Walters (2004)
FAO World Food Model (FAO)	multi-market partial equilibrium	FAO2050	agricultural production, demand, prices and trade	population, GDP, agric. productivity	115 regions	14 agric. commodities	www.fao.org
WATERSIM (IWMI/IFPRI)	linked multi-market partial equilibrium and hydrology	CAWMA	agricultural production, demand, prices and trade, water use	population, GDP, agric. productivity,	282 sub-basins	32 food commodities	de Fraiture *et al*. (2007)
BLS (IIASA)	computable general equilibrium	Parry *et al*. (2004)	agricultural production, demand, prices and trade, GDP	population, productivity, climate	34 regions	10 production sectors (9 agric sectors)	Fisher *et al*. (1988)
Agrobiom (INRA/CIRAD)	biomass	Agrimonde	calorie balances	population, agric. productivity, land use	149 regions	5 biomass categories	Chaumet *et al*. (2009) (outline only)

The studies of the MA and Parry *et al*. (2004) adopt general equilibrium representations of global production, consumption and trade, in which sectoral and economy-wide variables including aggregate income, factor prices and real exchange rates are simultaneously determined in an internally consistent manner. In contrast, the World Agriculture Towards 2030/2050 and CAWMA scenarios are based on a partial equilibrium approach, which treats global markets for individual agricultural commodities one by one in isolation from each other. In these models, regional demand and regional supply for each agricultural commodity is a function of its market price for given levels of income and given productivity drivers, and the model solves endogenously for the world market price that equates global supply and demand. The partial-analytic approach ignores economy-wide constraints including budget constraints on the demand side, balance-of-payments constraints and aggregate land endowment constraints, as well as repercussions of shocks to agricultural markets on aggregate income. This simplifies the analysis considerably, but limits the domain of applicability of these partial-analytic models to scenarios in which major shocks that affect many agricultural commodities simultaneously do not occur. On the other hand, partial equilibrium multi-market models like the World Food Model, IMPACT and WATERSIM support a more detailed commodity disaggregation than global computable general equilibrium (CGE) models.

Among the five scenario exercises considered here, the MA scenario study employs the most complex and sophisticated modelling framework. Its centrepiece is the global integrated assessment model IMAGE, developed at the Dutch National Institute for Public Health and the Environment (RIVM). IMAGE is designed to capture interactions between economic activity, land use, greenhouse gas (GHG) emissions, climate, crop yields and other environmental variables. It includes a multi-region CGE model of global trade and production, a carbon-cycle module to calculate GHG emissions resulting from economic activity including energy and land use, a detailed land-use module and an atmosphere–ocean climate module that translates GHG emissions into climate outcomes. The model-determined temperature and precipitation outcomes in turn feed back into the performance of the economic system via agricultural productivity impacts.

For the purposes of the MA study IMAGE has been ‘soft-linked’ to a range of other simulation models (listed in table 1) to achieve a further downscaling of variables of interest. In soft-linked model ensembles, output variables from one model are used to inform the selection of values for the input variables or parameters of another model, but the different models are not formally merged—or hard-wired—into a single consistent simultaneous-equation system. Downscaling refers to the process of disaggregating variables towards a more detailed spatial or commodity classification scale. For instance, changes in crop yields owing to climate change predicted by IMAGE have been used to adjust the agricultural productivity parameters of the agricultural market model IMPACT, which features a finer disaggregation of crops by type and region than IMAGE. Similarly, the soft link with the integrated assessment model AIM provides downscaled results for the Asia-Pacific region. Changes in irrigation within IMPACT as well as the climate projections of IMAGE have been used as inputs for the WaterGAP hydrology and water-use model simulations to assess water stress.

Owing to the heterogeneity of scales, accounting methods and conceptual frameworks across different models, the soft-linking approach is associated with substantial problems in achieving consistency and is susceptible to error propagation. The scientific basis for linking models across disciplines and scales is still weak and requires specific attention in future research (Ewert *et al*. 2009). On the other hand, links can be based on established models and can exploit the embodied specialized knowledge from different disciplines rather than requiring new modelling work. As Böhringer & Löschel (2006) put it, these pragmatic advantages may outweigh to some degree impending deficiencies in overall consistency.

The Agribiom tool employed in the Agrimonde study endeavours to simulate regional supplies, uses and balances of physical food biomasses and their calorie equivalents without any attempt to determine market prices for agricultural commodities. Thus, the simulated outcomes may be achievable in a biophysical sense but are not necessarily viable in an economic sense.

As pointed out in §2*b*, the simulation results from any dynamic global simulation analysis for a long-term horizon of several decades are surrounded by numerous uncertainties—about the adequacy of the model structure to capture the key factors at work, about the presence of nonlinearities that entail tipping points beyond which fundamental change in systems behaviour occurs, about model parameters, and about the evolution of the main drivers of change in agricultural systems. Model outputs should not be misinterpreted as forecasts with well-defined confidence intervals. Rather they are meant to provide quantified insights about the complex interactions in a highly interdependent system and the potential general size order of effects, which cannot be obtained by qualitative and theoretical reasoning alone. The results are crucially contingent on the current state of scientific knowledge used in the course of the development and parameterization of the model components. For example, the skill of the climate model component in IMAGE is necessarily restricted by the current state of the art in global circulation modelling and hence precipitation is poorly represented, which in turn limits the accurate simulation of crop responses.

## 4. Case studies of food system scenarios

**Figure 1.**
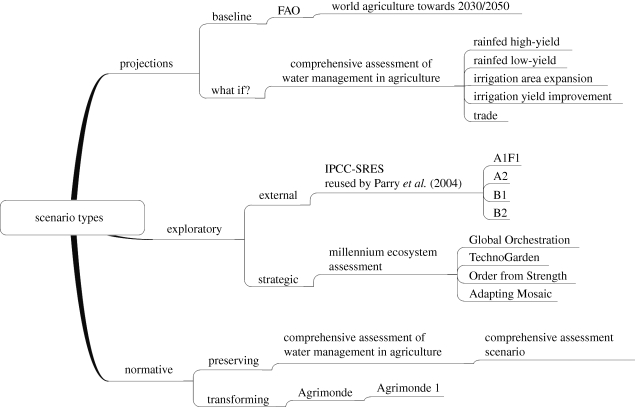
Classification of review studies based on scenario typology. Source: modified from Borjeson *et al*. (2005).

This review will adopt a conceptualization of the food system and its outcomes suggested by Ericksen (2008) where food system activities are linked to social welfare, food security and natural capital outcomes. Case studies have been chosen to illustrate our typology (figure 1).

### (a) Projections

#### (i) World agriculture towards 2030/2050

The UN Food and Agricultural Organization (FAO) produced a baseline projection of the food system to 2050 using its partial equilibrium, World Food Model (Alexandratos 2006). One of the main purposes of this scenario was to consider whether a revision by the UN in 2004 of its population growth projections could result in a Malthusian future.

In this future, growth in cereal productivity declines from 2.1 per cent per annum in 1961–2001 to 1.2 per cent per annum in 2001–2030 and then to 0.6 per cent in 2030–2050. However, this decline occurs alongside slowing population growth rates; and per capita consumption levels improve in developing countries to reach an average of 3070 kcal per capita by 2050. A peak in the population by the middle of the century is expected to ease the demands on natural capital from agricultural production. Reductions in absolute numbers of those malnourished are tempered by population growth but the proportion falls from 20.3 per cent in 1990/1992 to 3.9 per cent by 2050. Nevertheless, countries that increase their per capita consumption levels could still face a ‘double burden of malnutrition’ on healthcare systems if diets contain a higher proportion of fat, sugar and salt. Increasing demand in developing countries heightens import dependencies: but the market is projected to adapt autonomously, and developing world net exporters increasingly trade with developing world net importers. Growing competition among developing world producers to supply a relatively static market of developed world consumers leads to some price instability. The scenario was produced before the food price spike of 2007, which has been attributed partly to a rise in first-generation biofuel production (World Bank 2008). Although the implications for the food system of future energy prices are not fully explored in this baseline projection, there is foresight in its call for more analysis on the prospects of competition for land between food and fuel. Finally, there remain several countries, identified as vulnerable to food insecurity in this future, challenged by a deleterious confluence of high population growth rates, limited prospects for enabling economic growth, and low capacity for agricultural production.

#### (ii) Comprehensive assessment of water management in agriculture

**Figure 2.**
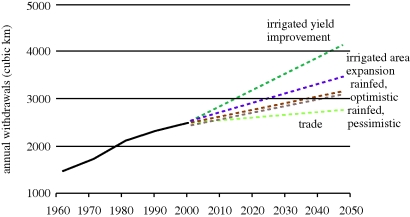
Global water withdrawals for agriculture based on CAWMA scenarios of alternative investment strategies. Source: de Fraiture *et al*. (2007). Reproduced with permission of Earthscan Ltd (http://www.earthscan.co.uk).

The CAWMA created five ‘what-if?’ projections to test the efficacy of alternative investment approaches to meet the projected food demand in 2050 (de Fraiture *et al*. 2007) (figure 2). The scenario narratives rely on outputs from WATERSIM, a quantitative model consisting of two integrated modules: a partial equilibrium framework based on the IMPACT model simulating food supply and demand, and a water balance and accounting framework simulating the supply and demand of water.

To meet the projected food demand, it has been estimated that water use for crops, or evapotranspiration, will have to increase by around 70–90% (de Fraiture *et al*. 2007). However, agriculture is likely to face competition from other sectors for freshwater; its use is more consumptive; withdrawals may not be accessible or sustainable; and pollution is increasing (Shiklomanov 2000). Although equipped irrigated areas have more than doubled since 1960, more than half of agricultural production still comes from rainfed agriculture, which is inherently uncertain.

In the ‘rainfed optimistic’ scenario increasing concerns about the high cost and environmental impacts of large-scale irrigation provoke a step-change, whereby there is no expansion in the irrigation area for crop production. Instead there is a focus on rural, poor smallholders in rainfed areas. Institutional reform encourages farm-level adoption of recommended production practices including *in situ* water management and harvesting techniques. Around 80 per cent of exploitable yield gaps are assumed to be bridged by 2050. The projections of this scenario suggest that there is at least the potential of rainfed agriculture to meet additional food requirements globally.

The risks in a predominantly rainfed strategy are demonstrated in the ‘rainfed pessimistic’ scenario. In this scenario, only 20 per cent of exploitable yield gaps are bridged by 2050, mostly as a consequence of slow rates of adoption of recommended production practices. The rainfed area must increase by 53 per cent to meet future food demands; such expansion is feasible but there may be negative environmental consequences. Countries without potential to expand rainfed areas must increase food imports; and the volume of global food trade necessarily increases. Lower levels of food availability and accessibility in poorer countries exacerbate food insecurity, which is adjudged to be highest in this particular scenario.

Alternatively, in the ‘irrigation expansion’ scenario there is an emphasis on food self-sufficiency and improved access to agricultural water for more people, particularly in Asia and Sub-Saharan Africa. Yet, expanding the irrigated area by 33 per cent meets less than 25 per cent of additional global food demand. Furthermore, the costs of such expansion are substantial—estimated at around US$400 billion to expand the harvested area; with additional costs to build supporting infrastructure and create institutional capacity to manage irrigation schemes. Although food security improves and rural incomes are enhanced, pressure on freshwater resources increases. The number of people experiencing physical water scarcity increases from 1.2 billion to 2.6 billion in 2050. There is increased competition among sectors and trans-boundary conflicts intensify. In several basins, minimum environmental flow requirements are not satisfied, implying adverse environmental impacts of withdrawals on ecosystems and fisheries.

Many irrigation schemes, particularly in South Asia, perform below their potential and there are opportunities for improving water productivity. The ‘irrigation yield improvement’ scenario assumes that 75–80% of exploitable yield gaps are bridged in coming decades from a combination of institutional reform, better motivation of farmers and water managers to improve productivity of land and water, and improved water allocation mechanisms among competing actors. Improving irrigated yields contributes around 50 per cent of increased global food demand by 2050; there is also a 9 per cent expansion of irrigated area globally. Irrigated diversions increase by 32 per cent but a larger amount of diverted water is used beneficially by crops, livestock or other productive processes. Investment costs are again substantial and are estimated at around US$300 billion.

The efficacies of these alternative strategies are dependent on regional agro-ecological capability and capacity (Fisher *et al*. 2002), and outcomes for regions vary considerably. In the ‘trade’ scenario countries with capability and capacity export to countries that do not. The logic in this scenario recognizes an increasing awareness of the concept of virtual water trade (Allan 1998; Hoekstra & Chapagain 2008) as well as the relatively modest volumes of trade in developing countries. Cereal trade, for example, relieves pressure on irrigation water because major grain exporters in the USA, Canada, Argentina and France produce grain in highly productive rainfed conditions. Thus, trade has the potential to mitigate water scarcity and reduce environmental degradation. Increases in food demand can be satisfied through international trade without worsening water scarcity or requiring additional costly irrigation infrastructure. However, trade alone will not solve structural problems of water scarcity; and poor water-scarce countries may not be able to afford to import large amounts of agricultural commodities without foreign currency from exports. Countries struggling with food insecurity may be wary of depending on imports to satisfy basic food needs, especially after the recent food price spike. The inherently political nature of the food system also suggests that it is simplistic to assume that freer international trade is readily achievable even if it is considered by many to be beneficial to food system outcomes. Trade, furthermore, requires energy and recent spikes in oil prices have resulted in de-globalization hypotheses (Rubin 2009).

### (b) Exploratory scenarios

**Figure 3.**
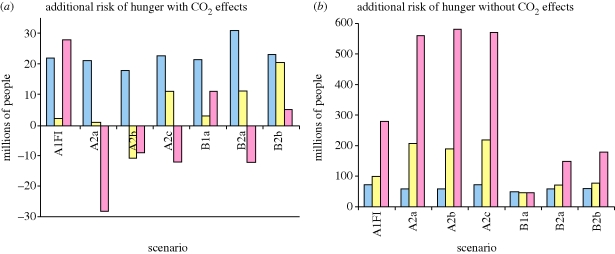
Additional millions of people at risk under seven SRES scenarios with and without CO_2_ fertilization effects, relative to a reference scenario with no climate change. Source: Parry *et al*. (2004). Blue bars, 2020; yellow bars, 2050; pink bars, 2080.

Parry *et al*. (2004) explored the impact of climate change on food security outcomes to 2080. Socio-economic scenarios (A1FI, A2, B1, B2), previously produced by the Intergovernmental Panel on Climate Change were reused (Arnell *et al*. 2004). A modelling framework, based on a general equilibrium approach, was created to estimate the response of cereal yields to simulated climate change based on these scenarios, and then to quantify the implications for cereal production, prices and risk of hunger (figure 3). Uncertainty in the socio-economic dimension of the food system (e.g. population and GDP growth) is managed with scenarios, whereas uncertainty in the biophysical dimensions (cereal productivity growth) is managed using modelling. Although this study resembles ‘what-if’ projections, it augments a set of external exploratory scenarios.

The A1FI scenario is a globalized future with very rapid economic growth and greater distribution of income between regions. Population growth is low and, similarly to the FAO baseline projection, peaks by mid-century. The energy system in this future is fossil fuel-intensive, global temperatures are the highest and cereal yields suffer most, especially in Africa and parts of Asia. Assuming no CO_2_ fertilization effects, aggregate cereal yields worldwide are depressed by roughly 10 per cent in 2050 compared with a reference scenario, there are large price increases, and an additional 100 million people may be at risk of hunger. With CO_2_ fertilization effects, many areas witness yield increases, apart from Africa, which is unable to counter a 20 per cent reduction. The effect of carbon fertilization limits rises in prices to around 10 per cent and the additional risk of hunger is hugely reduced.

A2 is a heterogeneous world where there is more self-reliance and preservation of local identities. Population is higher and economic growth less rapid than in A1FI. Although there is an increasing divergence in cereal yields between developed and developing countries in all the scenarios, the differences are greatest in this scenario. In particular, yields dramatically decrease in developing countries with regional temperature increases and precipitation decreases. Although the impact on production upto 2050 is similar to A1FI, prices are higher, and with a larger and poorer population the additional number of people at risk of hunger is greater. Without CO_2_ fertilization effects, around 200 million people are additionally at risk of hunger by 2050 and there are almost 6000 million by 2080.

B1 is a globalized future with the same low population as A1FI, but economic development follows a more environmentally sustainable pathway. Global temperatures in B1 are the coolest of the IPCC scenarios and cereal production decreases without CO_2_ fertilization effects are around half that of A1F1 and A2. The CO_2_ fertilization effect is less significant in this future because of the lower levels of CO_2_ concentrations in the atmosphere. Including the CO_2_ fertilization effect limits production decreases; but these reductions are smaller than in the A1FI and A2 scenarios because B1 has less CO_2_ in its atmosphere. Price increases are the lowest in the scenarios with or without CO_2_ fertilization effects at just over 10 per cent and just under 50 per cent, respectively. Without CO_2_ fertilization effects, the additional people at risk of hunger in 2050 and 2080 are considerably less than in A1FI and A2 futures, which are dominated by economic growth.

In contrast to B1, in the B2 world there is an emphasis on local rather than global solutions to economic, social and environmental sustainability. Population increases but at a rate lower than A2. Economic growth in this more regionalized world is also moderate. Food security outcomes such as production, prices and additional people at risk of hunger are a little worse than in B1 but better than in A1FI and A2.

Parry *et al*. (2004) find that, based on IPCC scenarios, it will be possible to feed a growing world population in 2050. While climate change appears likely to widen the difference in cereal yields between developed and developing countries, global trade prevents negative food security outcomes. However, regional outcomes will vary, particularly in Africa, Latin America and parts of Asia, and the number of additional people at risk of hunger may increase, especially to 2080. CO_2_ fertilization effects are likely to be an important determinant of future food security outcomes in 2050; but if such effects are based on experimental results in either controlled environmental conditions or optimal conditions, the benefits for low-input, stressed environments may be over-estimated (Long *et al*. 2006). Results also suggest that the major climate stressors for agricultural production could lie from 2050 to 2080 (figure 3).

#### (i) Millennium ecosystem assessment

**Figure 4.**
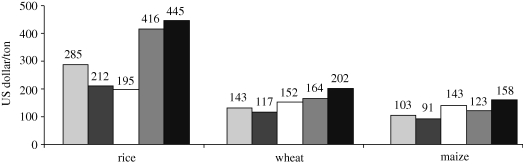
International cereal prices in the millennium ecosystem assessment (MA) scenarios in 2050. Source: Carpenter *et al*. (2005). Light grey bars, 1997; dark grey bars, TechnoGarden; white bars, Global Orchestration; medium grey bar, Order from Strength; black bars, Adapting Mosaic. Source: Millennium Ecosystem Assessment 2005 *Ecosystems and human well-being: scenarios.* Reproduced by permission of Island Press, Washington, DC.

The main objectives of the scenario study conducted as part of the 2005 MA are ‘to assess future changes in world ecosystems and resulting ecosystem services over the next 50 years and beyond, to assess the consequences of these changes for human well-being, and to inform decisions-makers at various scales about these potential developments and possible response strategies and policies to adapt to or mitigate these changes’ (Carpenter *et al*. 2005, p. 450). The four MA scenarios are framed in terms of contrasting evolutions of governance structures for international cooperation and trade (globalized versus regionalized) and cooperation and contrasting approaches towards ecosystem management (pro-active versus reactive). The approach to scenario development uses an iterative process of qualitative storyline development and quantitative modelling in order to capture aspects of ecosystem services that are quantifiable as well as those that are difficult or impossible to express in quantitative terms. The scenarios can be classified using our typology as exploratory and strategic. In conception, the results of the quantitative simulation models are meant to ensure the consistency of the storylines (figure 4). However, in practice, time constraints limited the number of iterations and the MA scenario report candidly admits the presence of remaining inconsistencies between storyline narratives and simulation results.

In all four scenarios global per capita food production in 2050 is higher than in the 2000 base. Thus, none of the futures presented is a classic Malthusian scenario (Willenbockel 2009). However, the global average masks considerable variation across regions within the individual scenarios.

Under the Global Orchestration (GO) scenario, which is characterized by global trade liberalization, global cooperation and a reactive approach towards environmental management, by 2050 agricultural output in both developed and developing regions is mostly produced on large highly mechanized farms. Low-intensity farming continues only as a lifestyle choice and on marginal lands in least developed areas. Despite this intensification, crop area expands globally as the share of meat in people's diets increases with growing prosperity, which in turn raises the demand for animal feed. Around 50 per cent of sub-Saharan Africa's forests are envisaged to disappear towards 2050. Growth in per capita calorie availability is highest among the four scenarios, and child malnourishment drops to around 40 per cent of current levels.

In the TechnoGarden (TG) scenario, a proactive technology- and market-based approach to ecosystems fosters a rapid transformation of agriculture across the globe. In developed regions, the assignment of property rights generates incentives for farmers to dedicate land increasingly to the provision of multiple ecosystem services. The elimination of agricultural trade barriers attracts investments from agri-business and supermarket chains into Latin American, African and Eastern European agriculture and leads to agricultural intensification in combination with an increasing development and adoption of locally adapted genetically modified crops in these regions. Indeed, sub-Saharan Africa is envisaged to turn into ‘one of the globe's ‘breadbaskets’ with some of the cleanest cities and most rational land use in the world’ (Carpenter *et al*. 2005, p. 259). Calorie consumption levels and child malnourishment are similar to the GO scenario.

The Adapting Mosaic (AM) scenario is a future with an emphasis on local approaches and local learning to the improvement of ecosystem services and with diverse outcomes across regions. Under AM, the WTO Doha Round trade liberalization negotiations break down and climate change mitigation as a globally coordinated effort disappears from the policy agenda. Global increases in calorie availability are very low compared with GO and TG.

Food system outcomes are worst under the Order from Strength (OS) scenario, which combines a reactive approach to ecosystem stresses with high trade barriers and low levels of global cooperation. Per capita food availability in 2050 reaches only around 80 per cent of GO levels. OS is the only MA scenario with rising child malnutrition. Owing to insufficient investment in yield improvements, production growth necessitates significant crop area expansion in both developed and developing regions. The outlook for sub-Saharan Africa is particularly concerning: OS envisages a significant decline in farm output exacerbated by climate change impacts, and widespread food insecurity as a trigger of mass migration from southern to West and East Africa, leading to social unrest and civil war in the latter regions.

### (c) Normative scenarios

#### (i) Comprehensive assessment scenario

The CAWMA also developed a preferred future of optimistic investment approaches to meet the target of feeding a global population of 9 million in 2050 (de Fraiture *et al*. 2007). In scenario analysis preferred futures are often referred to as a ‘fifth scenario’.

The findings from the five scenarios developed previously (rainfed optimistic, rainfed pessimistic, irrigation expansion, irrigation yield improvement, trade) strongly favour a portfolio approach to investment that is customized for each region. In South Asia, the emphasis is on irrigation yield improvement, with limitations placed on new irrigation development so that there is a focus on poverty reduction of smallholders and groundwater resources are protected. On the other hand, in sub-Saharan Africa, the emphasis is on improving the performance of rainfed agriculture. Smallholders concentrate on producing labour-intensive crops for local markets. Physical and institutional infrastructure enables rural growth and poverty reduction, and eventually with urbanization and diversification, farm sizes and incomes increase. There is also an increase in the irrigated area by around 80 per cent to support production of high-value cash crops such as sugar, cotton and fruit. For the Middle East and North Africa freshwater withdrawals are subject to increased regulation and there is a switch from irrigated cereal crops to higher value fruit and vegetables. East Asia improves existing irrigation productivity and with the integration of fisheries in paddy production, aquaculture production increases. China, in particular, regulates environmental flows more carefully and becomes a major grain importer. There is an expansion of cultivated areas in Eastern Europe, Central Asia and Latin America, mostly for rainfed production. Latin America increases exports of sugar, soya beans and biofuels. In OECD countries aquatic ecosystem services are restored and agricultural exports fall with subsidy reform. The global average rainfed cereal yield increases by 58 per cent and rainfed water productivity improves by 31 per cent. For irrigated yields the increase is 55 per cent and water productivity improves by 38 per cent. Globally, harvested areas increase by 14 per cent, although much of the increase in the harvested irrigated area comes from cropping intensity rather than from expansion. Negative impacts on terrestrial ecosystems are mitigated by regulation. Freshwater withdrawals by agriculture increase by only 13 per cent in 2050 in this normative, preserving future.

#### (ii) Agrimonde 1

The Agrimonde project, jointly initiated by the Institut National de la Recherche Agronomique and the Centre de Coopération Internationale en Recherche Agronomique, created a mostly qualitative scenario of a sustainable food system that feeds a global population of 9 billion people in 2050. It uses a basic quantitative tool called Agribiom to simulate regional supplies, uses and balances of physical food biomasses and their calorie equivalents but does not attempt to determine market prices for agricultural commodities (Chaumet *et al*. 2009). This future, entitled Agrimonde 1, was inspired by a book that proposed a sustainability scenario for the food system driven by a ‘doubly green revolution’ (Griffon 2006). The normative target is, thus, that in 2050 the world has developed a sustainable food system. In fact, it is assumed provocatively that in each region there is an equalization of consumption to an average of 3000 kcal per person per day in 2050.

In the late 2010s, increasing instances of food crises threaten social and political stability. Values converge among actors and the concept of a sustainable food system is pursued following ‘hunger riots'. A globalized community of practice evolves to manage ecosystem services and there are limits on proprietary intellectual property. Climate change has driven technological development in agriculture towards an ecological intensification that is sufficiently productive yet minimizes environmental externalities for soil, water and biodiversity. Greater biodiversity is assumed to improve system resilience. Such paradigms for sustainable agriculture have been advocated for developing countries (Pretty *et al*. 2006). An energy crisis in the 2020s provokes a step-change in the energy system towards decentralization of production. By 2050, there is global governance to prevent distorting policies and to intervene in the management of reserve stocks in order to protect import-dependent countries. Markets are regulated to prevent price volatility. There are also national and regional strategies integrated at different layers of power devoted to food security. Greater investments in infrastructure and social services have been partly made possible by improved income from rural areas. The industrial agricultural model, though initially dominant, merges with more local food and agricultural systems, especially in developing countries. There is a lower proportion of processed to raw products; and regulations impose greater accountability on companies to support nutritional objectives.

In OECD countries, reductions in kilocalorie per capita consumption are driven by less waste, better nutrition policy and behaviour change; in sub-Saharan Africa, increases are driven by sustainable economic development. Latin America and sub-Saharan Africa successfully exploit supply-side yield gaps where agro-ecological capability and capacity are available. Countries in the former Soviet Union also exploit yield gaps but on land with less potential. Yield gaps between the least productive and the most productive have narrowed. A new generation of biofuels has also emerged by 2050. The world's total crop area (food and non-food) is extended by 39 per cent to 2050 with new croplands mainly in Latin America and sub-Saharan Africa. Pasture is the land cover mostly converted because of pressures to conserve forests. The irrigated area is static in all regions except sub-Saharan Africa where it has doubled, and in Asia where there has been a slight increase. Three regions have aggregate import dependencies; Asia has to import calories for animal feed; and it is necessary for the Middle East, North Africa and sub-Saharan Africa to import to satisfy food demand. Three regions have surpluses—OECD countries, Latin America and the former Soviet Union.

### (d) Discussion of case studies

**Figure 5.**
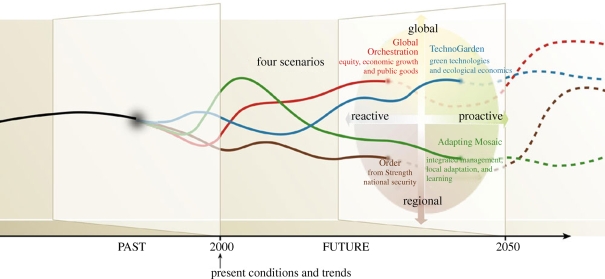
Axes of the MA scenarios. Source: Carpenter *et al*. (2005). Source: Millennium Ecosystem Assessment 2005 *Ecosystems and human well-being: scenarios.* Reproduced by permission of Island Press, Washington, DC.

The challenge of communicating multiple futures of complex systems has led to a preference towards scenario axes of two relatively independent, high impact, highly uncertain dimensions of uncertainty (Alcamo 2001) (figure 5). Rigorous and transparent management of uncertainty is necessary to judge the adequacy of any model to be a proxy to the future system (Wack 1985*a*; Rotmans & van Asselt 2001). Nevertheless, quantitative food system models are valuable in managing existing knowledge on system behaviour and ensuring the credibility of qualitative stories.

Wack (1985*a*) argues that the most important part of the scenario analysis process is to challenge the mental maps that actors use to navigate the future of the system of interest. Projections based solely on a model of the existing system may help to point towards sensitivities in the system and highlight new policy areas worthy of present attention but they are less suitable to manage uncertainty over long-term horizons. Indeed, the FAO baseline projection acknowledges the need for greater analysis of the impact of rising energy prices on food system structure. The CAWMA is an interesting example of the potential of multiple scenarios to simplify policy challenges rather than complicate them (Schoemaker 1993). Its five scenarios point compellingly to a normative preferred future for the food system. Regardless of this ‘fifth scenario’ these strategies can only ameliorate the increase in freshwater withdrawals that will be required to feed the global population in 2050. Variation in regional agro-ecological capability and capacity, and diversity in agricultural systems, suggest that a strategic portfolio of policy responses will be necessary. Input assumptions for highly significant socio-economic drivers of change such as GDP growth are held constant across the scenarios to test the sensitivity of the system to alternative investment strategies; and although this may have been suitable for the purpose of the exercise in question, this method would not have been appropriate for exploratory scenario analysis.

There are no Malthusian scenarios in Parry *et al*. (2004), even if food security outcomes in the A2 scenario include an estimated increase in the number of people at risk of hunger to 600 million by 2080. The limitations of the analysis are transparently acknowledged and highlight areas where innovation is necessary in biophysical modelling. For example, crop yield change estimates assume pests and diseases are controlled; flooding is not simulated in the crop models; assumptions of farm-level adaptation are based on current technology; and hydrological processes are simplified because of the resolution of the climate simulations. The effect of CO_2_ fertilization on yields is an important ‘known unknown’. It should also be noted that where once the A2 scenario was considered to be an extreme future, it has increasingly begun to be viewed as ‘business as usual’ (Nelson *et al*. 2009).

Exploratory scenarios may be the most suitable scenario type for managing uncertainty in the food system over long-term horizons to 2050 but the development of such scenarios requires significantly more resources than projections (Willenbockel 2009). Interestingly, exploratory scenarios from previous exercises that include analysis of food system are homogeneous and group into scenario families with similar food security outcomes. Cumming *et al*. (2005) propose five scenario families for integrated environmental assessments. In ‘market forces’, economic growth is the overriding aim of the system and as a consequence there are negative environmental externalities; values are broadly individualistic. The ‘reformed market’ uses hierarchical governance to address such externalities with regulation at the expense of some economic growth. A disconnected world of ‘higher fences’ may be the result of de-globalization if protectionism rises and trade volume falls in response to anxiety and fatalism about the future. The ‘values change’ family of scenarios is characterized by convergence towards a more sustainable and egalitarian society. Lastly, regionalization and localism may produce a ‘multipolar world’.

For three recent integrated assessments that provide a reasonable fit to these families, food security outcomes are similar (Parry *et al*. 2004; Carpenter *et al*. 2005; UNEP 2007). Global aggregate food availability and accessibility outcomes are broadly similar in the ‘market forces’ and ‘reformed market’ scenario family with significant reductions in malnourishment; at a regional scale sub-Saharan Africa and Asia remain the regions at most risk of hunger. However, food security outcomes may worsen beyond 2050 in ‘market forces’ as negative environmental externalities accumulate. The ‘values change’ scenario produces the most positive food security outcomes at global and regional scales because this is a more equitable future, with positive economic convergence between regions, and livelihoods that are increasingly sustained by nature's income rather than from erosion of its capital. The ‘higher fences’ scenario family produces noticeably negative outcomes at global and regional scales as a consequence of protectionist trade, which limits food availability, and low economic growth, which reduces food accessibility. Negative environmental externalities are especially severe as agro-ecological capabilities are stretched beyond appropriate limits. The full implications of climate change for the food system are not yet examined in these case studies because of technical, methodological and epistemological uncertainties. Nevertheless, it is expected to challenge the adaptive capacity of agriculture production in the developing world by 2050 (Parry *et al*. 2004; Nelson *et al*. 2009). If climate change widens the difference in yields between developed and developing countries in the future, such a divergence in outcomes may be exacerbated by existing yield gaps in the present. If fences are erected—politically, economically or technologically—food security outcomes for vulnerable regions in this future are very worrying.

Agrimonde 1 provides the narrative of a pathway towards feeding the global population healthily and sustainably, but it is not able to underpin its analysis with a credible quantitative simulation of the food system. It is unsurprising that this scenario does not use model simulation outputs. Food system models simulate the future based on the past, and if the food system is expected to profoundly transform, as it does in this scenario, a quantitative proxy for the existing system is less valid. It is a scenario that deliberately challenges the mental maps of food system actors, not least in its assumption of an equalization in food demand and in its expectation of extensification. According to the internal logic of the scenario, a world with a sustainable food system is still vulnerable to negative food security outcomes. Moreover, the ‘values change’ scenario family, the step-changes required to produce a paradigm shift to a sustainable food system in 2050 are non-trivial. Multiple scenarios are, therefore, recommended for food system actors to prepare for the future with strategies that adequately hedge against uncertainty (Lempert *et al*. 2006).

Finally, if there is one conclusion that can be drawn across this diverse selection of case studies, it is that international trade will be a crucial determinant of food system outcomes, both for food security and sustainability. Yet, both the general and partial equilibrium modelling approaches have a tendency to smooth outcomes, based on a sequence of equilibria, which means that potential trade shocks and resulting discontinuities in the food system are difficult to simulate.

## 5. Challenges for food system scenario analysis and modelling

### (a) Challenges for scenario analysis

Scenarios are not predictions; and scenario analysis is arguably at its most powerful as a vehicle for experiential learning (Wack 1985*a*). Alcamo (2001) suggests that integrated environmental assessments employing qualitative scenarios analysis and quantitative modelling may influence policy-makers by managing knowledge in a way that is more communicable. Yet, there is a paucity of research on the impact of such assessments on system actors. An evaluation of the MA found ‘little evidence so far that the MA has had a significant direct impact on policy formulation and decision-making, especially in developing countries’ (Wells *et al*. 2006, p. 38). For environmental assessments more generally, Mitchell *et al*. (2006, p. 324) find that the nature of the process of knowledge co-production among stakeholders is a stronger determinant of influence than final outputs. For scenario analysis in particular, stakeholder participation is crucial (van der Heijden 2005, p. 220). Knowledge co-production may be impeded if scenario analysis is not sufficiently participatory or if the modelling process used to underpin narratives is not accessible.

Garb *et al*. (2008) highlight a social divide between scenario developers and users that results in a ‘clumsy hand-off’ of learning. Drivers of change affect the food system at global, regional, national and local scales (Hazell & Wood 2008). Food system actors also interact with the system at different scales and in a variety of ways. Although scenario analysis is necessary at the global scale, participatory processes with key stakeholders at other geographical scales may increase the quality of scenario analysis and improve its impact (Zurek & Henrichs 2007). Alternatively, in circumstances where this is not feasible, improving the transparency of the scenario and modelling process may be a pragmatic compromise to encourage engagement with other food system actors (Parson 2008; table 2). The process for developing a new generation of normative climate scenarios builds on some of these principles and may offer a useful way forward (Moss *et al*. 2010).

**Table 2.** Selected driver assumptions to 2050 from case studies. CAWMA assumes GDP growth from the MA TechnoGarden scenario. Agrimonde 1 crop area growth includes non-food crops. Some figures are annualized to aid comparison; n.a. means figures were not derived for or by the modelling framework or were not published. Source: Parry *et al*. (2004); Carpenter *et al*. (2005); Alexandratos (2006); de Fraiture *et al*. (2007); Chaumet *et al*. (2009).

scenario exercise	scenario	population in 2050 (in billions)	GDP growth to 2050 (per annum) %	aggregate food demand in 2050 (kcal per person per day)	cereal productivity growth to 2050 (per annum) %	crop area increase (per annum) %
FAO 2050 (base year 1999/01)	FAO 2050	8.9	3.1	3130	0.9	n.a.
CAWMA (base year 2000)	rainfed—high yield	8.9	2.2	2970	1.4 (rainfed)	0.14 (rainfed)
					0.7 (irrigated)	0 (irrigated)
	rainfed—low yield				0.4 (rainfed)	1.06 (rainfed)
					0.6 (irrigated)	0 (irrigated)
	irrigation—area expansion				0.4 (rainfed)	0.56 (rainfed)
					0.7 (irrigated)	0.66 (irrigated)
	irrigation—yield improvement				0.4 (rainfed)	0.66 (rainfed)
					1.5 (irrigated)	0.18 (irrigated)
	trade				1.2 (rainfed)	0.44 (rainfed)
					0.7 (irrigated)	0 (irrigated)
Parry *et al*. (2004) (base year 1990)	A1F1	8.7	3.6	n.a.	see Parry *et al*. (2004) for potential changes in yields	n.a.
	A2	11.3	2.3			
	B1	8.7	3.1			
	B2	9.3	2.8			
MA (base year 1997)	Global Orchestration	8.1	n.a.	3580	1.0	0.01
	TechnoGarden	8.8	n.a.	3270	∼0.9	0.11
	Adapting Mosaic	9.5	n.a.	2970	∼0.6	0.23
	Order from Strength	9.6	n.a.	3010	0.5	0.34
CAWMA (base year 2000)	CAWMA scenario	n.a.	n.a.	2970	1.1 (rainfed)	0.14 (rainfed)
					1.1 (irrigated)	0.32 (irrigated)
Agrimonde (base year 2000)	Agrimonde 1	9.1	n.a.	3000	n.a.	0.78

Wack (1985*a*,*b*) evaluates the impact of scenario analysis based on its ability to provoke decision-makers to reconsider and ultimately redraw the mental maps with which they navigate the future of a system. Schoemaker (1993), in an exploration of the psychological benefits of scenario analysis, concludes that scenario analysis can indeed expand thinking; but more empirical research is required into the ways in which scenarios can successfully alter the mental maps actors have of a system (Garb *et al*. 2008).

Thompson & Scoones (2009) challenge the worldviews with which the food system is envisaged. Basic narratives of growth, it is argued, have been over-emphasized, at the expense of more multi-dimensional narratives of adaptation. For long-term objectives of reducing poverty in the rural developing world and maintaining ecosystem services, alternative narratives of sustainable agriculture and participatory research and development are proposed. With notable exceptions such as the MA, the concept of sustainability across the social, economic, biophysical, political and institutional dimensions of the food system has been inadequately explored so far in integrated assessments, mostly for reasons of technical, methodological and epistemological uncertainty (Swart *et al*. 2004). Scenario analysis could be increasingly important in developing new worldviews of a food system that can feed a growing population healthily and sustainably in 2050.

### (b) Knowledge gaps and priorities for modelling research

It is widely acknowledged that more work on the validation of model components used in integrated assessment studies is required, yet existing data sources often do not provide a sufficient basis for an ex-post comparison of simulation results with historical observations. On the other hand, in the presence of climate change and potential nonlinearities and tipping points, there is a risk of over-calibrating models to past processes that might not necessarily be the processes driving future developments (Uthes *et al*. in press).

For modellers involved in integrated assessment, the availability, coverage, quality and accessibility of spatially explicit datasets for global crop production and trade, land use and hydrology are major concerns. In addition to primary data collection efforts, the development of an integrated data repository along with concordances between datasets that are based on different conceptual schemes and scales would be desirable. There is a need for scaling algorithms that ensure conceptual consistency of the data flow between model components that operate at different spatial, sectoral and temporal scales. Various up- and downscaling methods exist but knowledge about scaling in integrated assessment is still in a state of infancy and often lacks scientific rigour (Ewert *et al*. 2009). The EU SEAMLESS project may be seen as a promising initial effort in this direction.

The IPCC Fourth Assessment Report identifies a long list of knowledge gaps and associated research priorities related to climate change impacts on agricultural production (Easterling *et al*. 2007), which includes inter alia the need for (i) further free air CO_2_ enrichment (FACE) experiments on an expanded range of crops, pastures, forests and locations, especially crops of importance for the rural poor in developing countries; (ii) basic knowledge of pest, disease and weed response to elevated CO_2_ and climate change; (iii) a better representation of climate variability including extreme events at different temporal scales in crop models; (iv) new global simulation studies that incorporate new crop, forestry and livestock knowledge in models; (v) more research to identify highly vulnerable microenvironments and to provide economic coping strategies for the affected populations, since relatively moderate impacts of climate change on overall agro-ecological conditions are likely to mask much more severe climatic and economic vulnerability at the local level; and (vi) examination of a wider range of adaptation strategies and adaptation costs in modelling frameworks.
